# Assessing the Risk of Depression Tendency in Pregnancy and Puerperium during COVID-19 Pandemic in Poland

**DOI:** 10.3390/healthcare11142005

**Published:** 2023-07-12

**Authors:** Urszula Sioma-Markowska, Patrycja Krawczyk, Anna Brzęk

**Affiliations:** 1 Department of Nursing in Gynecology and Obstetrics, Faculty of Health Sciences in Katowice, Medical University of Silesia in Katowice, Medyków 12 Street, 40-752 Katowcie, Poland; 2 Department of Physiotherapy, Faculty of Health Sciences in Katowice, Medical University of Silesia in Katowice, Medyków 12 Street, 40-752 Katowcie, Poland

**Keywords:** pregnancy, postpartum, depression tendency, risk factors, EPDS, BDI-II, HADS

## Abstract

The aim of the study was to assess the risk and severity of depression tendency in pregnant and postpartum women and to determine the relative risk for selected psychosocial and obstetric variables. The study included 317 women in the perinatal period. The severity of depressive disorders was assessed using standard self-report scales: EPDS (Edinburgh Postnatal Depression Scale), BDI-II (Depression Inventory—Second Edition), and HADS (Hospital Anxiety and Depression Scale). High rates of depression tendency in women in the third trimester of pregnancy were reported in 48.05% of pregnant women (≥10 EPDS scores), 49.36% (≥12 BDI II scores), and 41.55% (≥8 HADS-D scores). In contrast, in women in the first week after delivery, respectively: 33.74%; 28.83%; 22.08%. In the EPDS assessment, 11.69% of pregnant women and 17.79% of postpartum women confirmed the presence of self-injurious thoughts. A woman’s diagnosis of depressive disorder before pregnancy increases the risk of postpartum depression tendency 3.35 times according to the EPDS, 3.51 times according to the BDI-II, and 4.89 times according to the HADS-D. Depressive disorders were significantly more common in pregnant women compared to women in the first week of postpartum. Systematic screening can identify risk factors for prenatal and postpartum depression.

## 1. Introduction

Depression is one of the most common mental disorders in the world today. According to a World Health Organization forecast, depression will be the factor most responsible for the Global Burden of Disease in 2030. The SARS-CoV-2 virus pandemic has brought many threats to the mental health of pregnant and postpartum women and increased levels of stress, anxiety, and feelings of insecurity. The impact of restrictions and social distance on mental health is definitely long-term. Findings by Bueno-Notivol J. et al. (2021) suggest that the rate of depression in the general population is seven times higher during the COVID-19 pandemic, with an estimated overall global prevalence of depressive disorders of about 3.44% [1].

It is important to realize that a woman’s depressive mood adversely affects the course of pregnancy, childbirth, and the postpartum period, and then is reflected in the daily functioning of primarily women in the role of mother, which also affects the behavior of the child and the surrounding family. An analysis of the literature shows that there is a comparable incidence of depression during pregnancy with postpartum depression.

Pregnancy depression is hardly accepted in Poland, although the range of its prevalence varies from 10–25% [2,3,4,5,6]. Hence, a great deal of scientific interest translates into the problem of depression related to pregnancy and childbirth. Additionally important is the fact of the sheer interest of pregnant women, who are beginning to increasingly consciously strive to preserve their mental health, especially during or immediately after such a difficult period for everyone as during the COVID-19 pandemic.

Depression in pregnant and postpartum women undiagnosed early can lead to serious disorders due to the fact that it begins stealthily and insidiously. Therefore, the National Institute of Clinical Excellence (NICE) guidelines (2007) clearly suggest that especially in pregnant and postpartum women, mental health disorders should be detected and diagnosed as soon as possible [7], and these tasks in the form of screening are redirected to obstetricians and midwives. The Regulation of the Minister of Health on the organizational standard of perinatal care, in force in Poland since 2019, indicates that the emotional state and risk of severity of depressive symptoms should be assessed three times: at 11–14 weeks of pregnancy (first trimester), at 33–37 weeks of pregnancy (third trimester), and after delivery during the midwife’s visit to the place of residence or stay of the mother and child.

There is little research on the effects of pregnancy on a woman’s mood. The research was conducted during the pandemic period—according to the title of the article—searching the database after restricting the articles to keywords: “pregnancy” and “mood” and “pandemic”, which found only 156 results and not all of them were related and consistent with the conducted own research. After reducing the conducted research to Poland, there were only four articles. Those that are available point to the fact that women experience maternity differently than assumed by as much as 90%. Emotional disorders occur much more frequently at this time than at other periods of a woman’s life [8,9]. A wide variety of mental disorders can appear, as the only episode strictly related to pregnancy, appearing perinatally, the first episode of an incipient disease process, or recurrence of chronic disorders [10]. Childbirth phobia was described by Hofberg and Brockington, among others [11]. Tokophobia is an incompletely understood phenomenon, so there is little data on its prevalence. It is estimated that tocophobia can affect up to 10% of pregnant women, of which 2% are extremely severe and require specialized care [12].

Poland has so far lacked a systemic solution for screening and treating a woman suffering from depression in the perinatal period. In the new standards of perinatal care, in which it is mandatory to assess a woman’s mental health for depression three times, no specific tool has been imposed [13].

Therefore, it should be emphasized that in obstetric practice, it is crucial to detect depressive disorders as early as possible, already during pregnancy, although it will certainly not be among the easiest, as evidenced by the detection rates of emotional disorders. It will be paramount to recognize when mood swings, irritability, or sleep disturbances should be considered outside the bounds of the norm typical for this period. Available studies emphasize the importance of quickly determining whether a pregnant woman is in a risk group and whether there are and what are the risk factors for emotional disorders in pregnancy and the postpartum period [14].

One of the more well-known tools for detecting mood disorders, including depression in the perinatal period, is the Edinburgh Postnatal Depression Scale (EPDS) [2]. The EPDS is a specific scale recommended by the National Institute of Health and Care Excellence (NICE) and the Polish Association of Midwives. Another tool applicable to screen for perinatal depression is a generic scale, the Beck Depression Inventory—Second Edition; (BDI-II) [15]. The Beck Depression Questionnaire is not a substitute for a medical examination, but it is an important indicator of the risk or severity of depressive symptoms in subjects. Our study also used the Hospital Anxiety and Depression Scale (HADS) as a simple and practical tool for measuring anxiety and depression [16]. Prenatal depression is one area that has not received a thorough study in Poland to date, which is why the authors were interested in this area.

The purpose of this study was to assess the risk and severity of depression in prenatal and postnatal women as measured by the Edinburgh Postnatal Depression Scale (EPDS), Depression Inventory—Second Edition (BDI-II), and Hospital Anxiety and Depression Scale (HADS), taking into account selected psychosocial and obstetric variables.

## 2. Materials and Methods

### 2.1. Studied Groups

Women over the age of 18, from the 28th week of pregnancy and up to seven days postpartum, were eligible for the study. Women with a history of confirmed depression treated in specialized clinics were excluded from the study. Only 317 completely completed questionnaires were considered for final analysis. The prospective, non-interventional study included 154 pregnant and 163 postpartum women, differing in age, material status, education, marital status, and obstetric history.

### 2.2. Methods

Three standardized self-assessment scales were used to assess the risk and severity of depressive disorders in perinatal women in both study groups: Edinburgh Postnatal Depression Scale (EPDS), Beck Depression Inventory—Second Edition (BDI-II), and Hospital Anxiety and Depression Scale (HADS). A proprietary survey questionnaire was used to collect sociodemographic and clinical (obstetric interview) data. The use of two self-report scales was intended to confront the obtained results of estimating the prevalence of depression in the studied groups of women and to obtain more reliable data, especially since the study was conducted during a pandemic period, which could have been a confounding factor, in addition to test whether both scales are useful tools for screening depression in pregnant and postpartum women. The survey was conducted in two ways: online and printed versions in obstetrics and neonatal wards of clinical and public hospitals in the Silesia area. The study was conducted during the COVID-19 pandemic from March 2021 to March 2022.

#### 2.2.1. Questionnaire

The EPDS contains 10 questions (range 0–30) relating to anhedonia, blaming, anxiety, fear, feeling overwhelmed, sadness-induced sleep problems, sadness, crying, and thoughts of self-harm. Each statement is scored on a scale of 0–3 points. The maximum number of points possible is 30, with as few as 10 points or marking an answer confirming a desire to self-harm taken as the lower limit for the likelihood of depression [15]. 

In the BDI-II, questions are closely matched to the criterion symptoms of depression. Conducting the Beck test promotes high accuracy in diagnosing a depressive episode according to accepted diagnostic criteria and accuracy in differentiating from other mental illnesses. The questionnaire consists of 21 statements with 4 selectable options, scored from 0–3 (no symptom—symptom of high severity), with scores ranging from 0 to 63. In interpreting the results of the test in our study, we used data based on the Polish normalization of the BDI-II by Łojek E and Stańczak J [17], adopting scores: 0–11—no depressive symptoms, 12–19—mild depressive symptoms, 20–25—moderate depressive symptoms, and 26–63—severe depressive symptoms.

The HADS consists of two independent scales assessing anxiety (HADS-A) and depression (HADS-D). Each scale contains seven statements relating to the subject’s current state. The subject assesses the severity of each trait by selecting from four responses. Each question in this Likert-type scale is scored from 0 to 3. Each survey has a maximum score of 21. Obtaining 0–7—points on each scale is considered normal, 8–10—discrete mood-related disorders, 11–14—moderate, and 15–21—severe [16,18]. 

#### 2.2.2. Statistical Methods

Data was collected and stored in accordance with the university’s legal regulations. The raw data was transcribed into an Excel database. The obtained results were statistically processed using the STATISTICA 12.PL program. Descriptive statistics were used in the evaluation of the obtained results by providing means, standard deviation, and medians. The tests used were: Chi2 with Yates correction and Mann-Whitney U. A statistical significance level of *p* ≤ 0.05 was assumed.

#### 2.2.3. Bioethical Committee

The study was obtained from the Bioethics Committee of the Silesian Medical University in Katowice to conduct the study (No PCN/CBN/0022/KB/283/21). Each of the self-assessment scales used contains questions or statements relating to the detection of depressive symptoms.

## 3. Results

The mean age of pregnant women in the study was 28.9 years, ±3.7 (Me 29.0), and of postpartum women 29.9 ± 5.6 (Me 29.0). The groups differed statistically significantly in place of residence (*p* = 0.00005), education (*p* < 0.00001), type of occupation (*p* = 0.02), and number of past pregnancies (*p* = 0.0001). Among pregnant women, first-pregnancy women predominated (68.83%), while in the group of obstetricians were multiparous women (53.37%). Pregnant women were significantly more likely to answer the question about psychiatric treatment before pregnancy (*p* = 0.0007) and the occurrence of prolonged periods of anxiety and sadness (*p* = 0.01). A total of 13.31% of respondents were diagnosed with depressive disorders before pregnancy. Lack of support from the husband/partner (family) was experienced by 10.39% of pregnant women and 4.91% in the postpartum period. In the EPDS test, 11.69% of pregnant women and 17.79% of postpartum women confirmed the presence of thoughts of self-harm. 

Analysis of the reliability of the psychometric scales used in the conducted research project showed high reliability as determined by the α-Cronbach internal consistency coefficient for these scales in both study groups, significantly higher than the required value of 0.600 taken as the threshold of acceptable reliability (Table 1).

**Figure 1:**
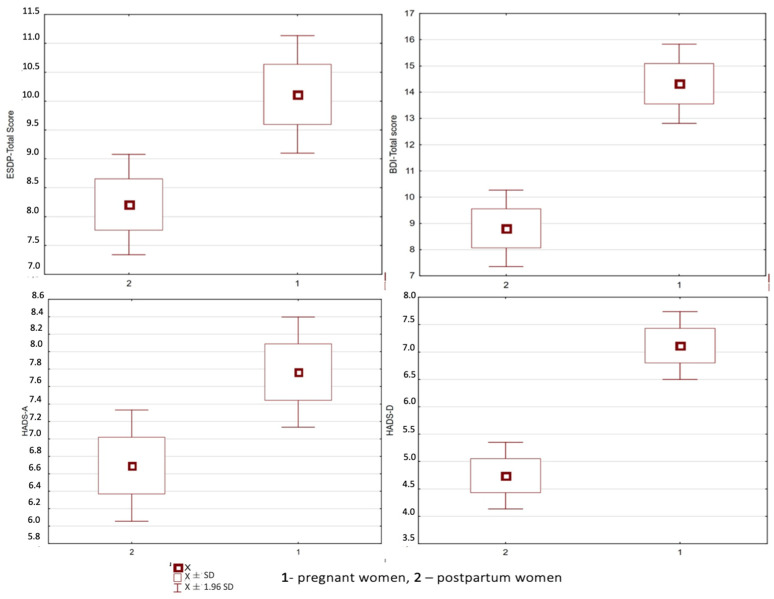
Index values (total scores) of psychometric scales obtained in groups of female respondents (X, SD).

A summary of the parameters of descriptive statistics of the indicators (number of total points) obtained in the questionnaires of the scales EPDS, BDI-II, and HADS, are presented in Figure 1. For each of the scales, statistically significant differences were obtained in the mean values of total scores obtained among the studied groups of women. Pregnant women were found to have higher values for each scale, especially for the BDI-II and HADS-D (*p* < 0.00001 in both cases). 

The distributions of indices (total scores) of the psychometric scales used relative to the threshold values in both groups of women studied are presented in Table 2. Only for the HADS-A scale, no statistically significant differences in distributions were shown (*p* = 0.22). The statistically strongest difference in distributions between the groups was found for the HADS-D scale (*p* = 0.0001). When assessed with this scale, only slightly more than half of the pregnant women (58.44%) showed no depressive traits (index up to 7 points) vs. 3/4 of the obstetrician group size (77.91%). For the EPDS scale, the difference in distributions was significant at *p* = 0.03, and for the BDI II scale, the significance level was *p* = 0.001.

Table 3 summarizes the relative risk (RR) values for the onset of disorders according to the threshold values of the scales in the group of pregnant women. Statistically significant relative risk values for the onset of depressive disorders (according to the EPDS) were obtained for high school education (RR = 1.95; *p* = 0.00005), poor or average financial situation (RR = 1.62; *p* = 0.002), for women who had prolonged periods of anxiety and sadness (RR = 1.49; *p* = 0.02), and for those women who could not count on support during pregnancy (RR = 2.01; *p* < 0.00001). 

A higher risk of depression tendency, according to the BDI-II scale indices, was shown for women with secondary education (RR = 1.84; *p* = 0.0001), with poor or moderate financial situation (RR = 1.56; *p* = 0.004), who were multiparous (RR = 1.44; *p* = 0.02), who had prolonged periods of anxiety and sadness (RR = 1.68; *p* = 0.0007), when diagnosed with a depressive disorder before pregnancy (RR = 1.79; *p* = 0.0002), and for those women who could not rely on support during pregnancy (RR = 1.95; *p* < 0.00001). A woman’s lack of support during pregnancy results in a 2.01 times greater risk of anxiety disorders as defined by HADS-A index values (RR = 2.01; *p* < 0.00001) and a 2.42 times greater risk of depressive disorders according to HADS-D (RR = 2.42; *p* < 0.00001). Lower education among pregnant women increases the risk of anxiety disorders according to HADS-A by 41% (RR = 1.41; *p* = 0.04) and depressive disorders according to HADS-D by 88% (RR = 1.88; *p* = 0.0008). 

Among non-working pregnant women, there is an increased risk of anxiety disorders by 1.58 times (RR = 1.58; *p* = 0.005) and depression tendency by 1.93 times according to HADS scales (RR = 1.93; *p* = 0.0002). Poor or average financial situations of women increases the risk of anxiety disorders by 44% (RR = 1.44; *p* = 0.02) and depressive disorders by 82% (RR = 1.82; *p* = 0.0008) (Table 4).

The relative risk values of the causal categories for those sociodemographic factors and clinical variables (pregnancy history) for which correlation with the distributions of psychometric scale indices was obtained in the group of postpartum women are shown in Table 4. 

For non-working women, there is an 84% higher risk of depressive disorders as determined by the EPSD scale (RR = 1.84; *p* = 0.004) and a 71% higher risk of depression tendency as determined by the BDI-II scale (RR = 1.71; *p* = 0.005). Women who were not in a relationship (maids) showed an increase of 1.76 times the risk of depression tendency on the BDI-II scale. Poor or average material situation for women was shown as a causal category for a 74% increase in the risk of depression tendency as determined by the ESDP scale (RR = 1.74; *p* = 0.01) and a 144% increase in the risk of depression tendency on the BDI-II scale (RR = 2.14; *p* = 0.0001). 

For multiparous women, there is an increase of 1.52 times the risk of anxiety disorders as determined by the HADS-Anxiety scale (RR = 1.52; *p* = 0.05). Among women who experienced complications during pregnancy, there is a 1.76-fold increase in postpartum risk of depression tendency as determined by the EPDS scale (RR = 1.76; *p* = 0.008) and a 2.65-fold increase in the risk of depression tendency as determined by the BDI-II scale (RR = 2.65; *p* = 0.00007).

Psychiatric treatment before pregnancy is a significant causal category for a 2.14-fold increase in the risk of anxiety disorders as determined by the HADS-A scale (RR = 2.14; *p* = 0.002). The occurrence of prolonged periods of anxiety and sadness in women results in a 2.60-fold increase in the risk of depression tendency on the EPDS scale, a 3.14-fold increase in depression tendency on the BDI-II scale, a 3.69-fold increase in depression tendency on the HADS-D scale, and a 2.23-fold increase in the risk of anxiety disorders on the HADS-A scale. Being diagnosed with a depressive disorder before pregnancy in a woman increases the risk of postpartum depression tendency by 3.35 times according to the EPDS, 3.51 times the risk of depression tendency according to the BDI-II, and 4.89 times the risk of depression tendency according to the HADS-D. This causal category also increases the risk of anxiety disorders by 2.49 times according to the HADS-A. Lack of postpartum support increases the risk of postpartum depression tendency according to the EPDS by 2.83 times, the risk of depression tendency according to the BDI-II by 2.84 times, and also increases the risk of anxiety disorders according to the HADS-A by 2.04 times. The relative risk value of this causal category for changes in the risk of depressive disorders according to HADS-D was found to be not statistically significantly different from 1 (RR = 1.14; *p* = 0.85) (Table 4).

## 4. Discussion

The main objective of the ongoing study was to estimate the risk of depressive disorders in the perinatal period, in a group of pregnant women (third trimester) and women in the first week of postpartum. 

The study was conducted during the COVID-19 pandemic period, which may affect the rate of diagnosed depressive disorders in pregnant and postpartum women. Experiencing traumatic situations, which include periods of lockdown and restrictions on social relationships, may increase vulnerability to the onset of symptoms of anxiety, restlessness, or depressive mood. The pandemic increased the level of fear in society [19]. The high rate of occurrence of depression tendencies in the first place may come as a surprise, the authors thought carefully. Research conducted during the pandemic period may have contributed to this. The increased risk of contracting the virus, the fear for an unborn child, or the fear of giving birth alone (without a partner present), or concerns about the lack of hospital beds may have influenced this, but also to an inadequate family environment, kind of information acquisition [20]. The lack of knowledge about the possibility of an end to the pandemic, as well as the emergence of new variants of the SARS-CoV-2 virus, is having a negative impact on the mental health of people around the world.

A self-reported study conducted using the EPDS, BDI-II, and HADS-D showed that depressive disorders were significantly more common in pregnant women compared to the postpartum period. High rates of depression tendency in women in the third trimester of pregnancy were reported in each scale. A high depression tendency score for women in the first postpartum week was found, but slightly lower than indicated above. 

A score of 14 points or more as measured by EPDS indicating the likelihood of severe depression tendency was obtained by 25.97% of pregnant women and 16.56% of postpartum women. In this regard, psychiatric consultation and therapeutic measures are indicated. The Polish Midwifery Association (2019) recommends that even with a score of 10 or higher, consultation with a specialist should be recommended. In the BDI-II score, a score of 26 points and above, suggesting a severe depressive episode, was obtained above 10% of pregnant and postpartum women. With such a high score, it is necessary to consult a psychiatrist and undertake intensive psychotherapy and/or inpatient treatment. Our own analysis does not correlate with the findings of Gavin et al. and Evans et al. that 1–6% of pregnant women experience a severe depressive episode [21], and 15–25% experience mild or moderate depression-related symptoms [3].

In the period before the COVID-19 pandemic, it was reported that prenatal depression affected women in the first trimester of pregnancy to a lesser extent (7.4%) compared to women in the second and third trimesters (12–12.8%, respectively) [22]. Another report cited 19% of pregnant women experience depression during pregnancy [23]. Women of reproductive age are at the highest risk for depression. The results of a study by Vesga-Lopez et. al. among American pregnant women shows that depression in pregnant women occurs with at least the same frequency as in non-pregnant women in the same age group. This study found that in the year prior to the analysis, the prevalence of depression tendency in pregnant and postpartum women was 8.4%, and bipolar disorder was 2.8%. In women who were not pregnant in the year preceding the study, depression was observed in 8.1% and bipolar disorder in 2.3% [24]. 

The self-reported results are three to four times higher, depending on the self-assessment scale used. There may be several reasons for this. First, the third trimester of pregnancy is a transitional period involving adaptation to emotional and physical changes, and women during this time have a poorer sense of well-being than during the other trimesters of pregnancy [25]. Secondly, the ongoing COVID-19 pandemic during the ongoing research project also significantly affected the results. An increased risk of depression and anxiety was also noted by Zhang et al., but they found no significant association between pandemic time and pre-partum scores. Women in the postpartum period were at increased risk (2.6 times more likely to score ≥13 on the Edinburgh Postpartum Depression Scale during the pandemic than before the pandemic) [26]. Different results were obtained by researchers from Israel, who concluded that postpartum women delivering during the COVID-19 pandemic have lower risk for depression compared to the comparison group of women not delivering during the pandemic [27]. In the Chiara et al. study, it was found that women delivering amidst the pandemic did not differ in their depressive and anxiety symptoms from their pre-pandemic scores and from pre-pandemic women. In the study population was a greater level of hypomanic symptoms during the COVID-19 period in response to the increased stress imposed by the pandemic [28].

The prevalence of postpartum depression in developed countries ranges from 5.2% to 74%, and in developing countries from 1.9% to as high as 82.1% [29]. In a Polish study (2019), a high rate of postpartum depression (≥12 points) as measured by the EPDS in the first postpartum week was achieved by 35% of women [30]. The authors were reflective of the results obtained, which were discussed. They took into account the period of the pandemic and the definite increase in the level of anxiety among the adult population. The study by Akgor et al. clearly confirmed our considerations. Women feared infection of their babies during childbirth. The fear of infection of the fetus during delivery revealed older age and anxiety as unique significant risk factors [31]. The high percentage of pregnant women experiencing depressive disorders implies the need for special mental health care for women during pregnancy and after delivery. During follow-up visits, assessing the mental state using simple and accessible scales will allow us to estimate the risk of depressive symptoms, qualify the group at risk of depression, and offer psychoeducation to this group.

Perinatal depression is not adequately diagnosed [32]. Polish guidelines for assessing the health status and severity of depressive symptoms in pregnancy and puerperium lack a recommended screening tool, so our study checked the reliability of the psychometric scales used. Our study proves the validity of the use of each of the self-assessment scales used in the study, both during pregnancy and the puerperium. The high α-Cronbach values obtained for all the tests used testify to the consistency of these scales. 

Many authors emphasize that pregnancy depression often precedes the onset of postpartum depression. Pregnancy can lead to a recurrence of symptoms, exacerbation of an already existing disorder, or trigger a first episode of depression [22,33]. Pereira et al., in addition to pointing out that during the COVID-19 pandemic, women in the postpartum period present greater depressive and anxious symptomatology, have additionally pointed to increased risk factors, as well [34]. Particularly vulnerable are high-risk pregnant women as pointed out by Kheirkhah et al. [35]. The rates of PPD increased for women who delivered during the first COVID-19 lockdown [36].

The results obtained, in relatively small subgroups of women surveyed, illustrate the scale of the problem. The women surveyed are young, of childbearing age, and largely without a history of burden. However, nearly one in five women surveyed had received psychiatric help before pregnancy. The risk and severity of depression increases in pregnant women who received psychiatric treatment before pregnancy [31].

In our study, we found a higher risk of pregnancy depression tendency (according to the BDI-II) in women who experienced prolonged periods of anxiety and sadness before pregnancy, when diagnosed with a depressive disorder, who could not count on the support of their husband/partner. A woman’s lack of support during pregnancy results in a 2.01-fold increased risk of anxiety disorders as defined by HADS-A index values and a two and a half times increased risk of depressive disorders, according to HADS-D. 

The findings of Cohen et al. indicate that the rate of recurrence of major depression in women who discontinued antidepressants during pregnancy was 68% compared to 26% in the group that continued therapy during pregnancy [37]. From the above data, it is clear that women who receive psychiatric treatment should receive care from both an obstetrician and a psychiatrist after becoming pregnant. When deciding whether to use medication in pregnant women receiving psychiatric treatment, it is important to keep in mind the high risk of relapse during pregnancy.

Previous episodes of emotional problems are also among the factors that contribute to the development of postpartum mood disorders. According to Kazmierczak et al., a history of depression in a previous pregnancy accounts for 35–50% of the risk of recurrence in a subsequent pregnancy [38]. Iracka et al. report that depressive disorders experienced during or before pregnancy are related to the occurrence of postpartum depression [39]. Dietz et al., in their study, found that 54.2% of patients who were diagnosed with depression during or before pregnancy also had postpartum depression [40]. Based on these results, one can see the great importance of assessing the risk of depression during pregnancy. The occurrence of possible mood disorders has long-term effects on the quality of life of the pregnant woman and the obstetrician.

Our own research showed that for multiparous women, the risk of anxiety disorders as determined by the HADS-A scale increases 1.52 times. Among women who experienced complications during pregnancy, there is a 1.76-fold increase in the risk of postpartum depression as determined by the ESDP scale and a 2.65-fold increase in the risk of depression as determined by the BDI-II scale. 

Psychiatric treatment before pregnancy is a significant causal category for increased risk of anxiety disorders as determined by the HADS-A scale. The occurrence of prolonged periods of anxiety and sadness in women before pregnancy results in an increased risk of postpartum depression tendency (2.60 times on the EPDS scale, 3.14 times on the BDI II scale, and 3.69 times depression tendency on the HADS-D scale). Being diagnosed with a depressive disorder before pregnancy increases the occurrence of postpartum depression tendency by 3.35 times according to the ESDP, 3.51 times according to the BDI II, and 4.89 times according to the HADS-D. Lack of support during the puerperium increases the risk of postpartum depression tendency by nearly three times (2.83) according to the ESDP and BDI-II.

The analyzed material showed that 15.58% of pregnant women and 6.13% of postpartum women experienced prolonged depressed mood before pregnancy (anxiety and sadness). These data confirm literature reports of many undiagnosed patients at high risk for depression tendency and anxiety disorders during pregnancy. It is stated that women experiencing COVID-19 infection during pregnancy were found to have greater anxiety and nervousness in the post-natal period compared to their COVID-19 negative counterparts [41]. Depression is one of the most common mental disorders, its chronic form is associated with severe functional impairment. The onset of pregnancy and impending childbirth can be perceived as a traumatic event and can potentially, in any woman, trigger lowered mood and depressive disorders. An additional stressor was the pandemic period. This confirms the importance of assessing the risk of depressive state and anxiety levels during pregnancy by medical personnel providing care to pregnant women. Women who required consultation with a psychologist or psychiatrist before conception often require continuation of this care during pregnancy.

The risk of depression is higher in unmarried women. In our study, we found a 1.76-fold increase in the risk of depression tendency in the postpartum period among unmarried women—maids (*p* = 0.05). This correlation may be due to insecurity in women who are single. A pregnant woman needs support from her partner/father of the child, who plays an important role during pregnancy. In their study, Podolska and Sipak-Szmigiel found that the marital status of pregnant women, especially cohabiting women, fundamentally affects the appearance of symptoms of perinatal depression and increases the risk of its occurrence [42]. An additional factor supporting the high risk of perinatal depression in women in single relationships is the fact that in Poland there is still a phenomenon of social stigmatization of women becoming pregnant before marriage [43]. However, it is important to view the results through the lens of the complexity of the biopsychosocial environment contributing to PPD as convicted Waschmann et al. [44].

Thoughts of self-harm were declared in the survey by 11.69% of pregnant women and 17.79% of postpartum women during the first week of confinement. Although no statistically significant differences in these distributions were shown, an affirmative answer to a question about the desire to self-harm is taken in the EPDS as a lower limit for the likelihood of postpartum depression. According to Cantwell R et al., the majority of suicides among pregnant and postpartum women (about 60%) occur in the 6 weeks before and 12 weeks after childbirth [45]. 

In our study, we found that depressed mood before pregnancy (prolonged periods of anxiety and sadness), diagnosed depression before pregnancy, and lack of husband/family support were significantly more likely to predispose to prenatal depression tendency, as well as postpartum depression. Psychiatric treatment before pregnancy, complicated pregnancy, and multiple births were more likely to predispose to postpartum depression tendency. The results of our study are consistent with reports by other researchers, who identified previous episodes of depression as the most significant factor in postpartum depression [31,46,47,48].

Of the psychosocial factors significantly contributing to both prenatal and postnatal depression tendency were barracking for work and poor or average financial situation. Lower education (high school) was significantly more likely to predispose to depression in pregnancy, while marital status—single—was significantly more likely to contribute to depression tendency in the postpartum period. The importance of psychosocial factors on the occurrence of depression tendency in the puerperium was also emphasized by other authors [47].

Our own and others’ studies [31,49] did not confirm the association of type of delivery with the occurrence of depressive disorders.

In conclusion, it should be emphasized that pregnancy is an emotionally significant event in a woman’s life. There are many individual factors on which the process of psychological and biological adaptation to pregnancy and motherhood depends. Modern society demands multiple roles in a woman’s life: wife/partner, mother, worker. The social context and designated roles are among the significant causes of mental health disorders. An additional factor in the elevated levels of stress and anxiety in pregnant women was the long pandemic. There is, therefore, a need for identification of potential risk and protective factors by implementing predictive models [50] and family education as new challenges to care for pregnant and postpartum women.

The self-assessment scales used in the study, EPDS, BDI-II, and HADS, are recommended for assessing well-being and depressive symptoms in the perinatal period, what can be considered a strength of the study; however, they all have some limitations in distinguishing depression tendency from other mental disorders. Our own research was done during the pandemic period, hence generalizing conclusions should be done with caution. In further research plans, it would be worthwhile to conduct a multicenter study that includes all time points for measuring mental status as defined in the standards of practice. 

## 5. Conclusions

Depressive disorders were significantly more common in pregnant women compared to women in the first week after delivery. There were no significant differences in psychosocial and clinical factors differentiating the risk of prenatal and postpartum depression tendency. The high α-Cronbach’s reliability coefficients for the EPDS, BDI-II, and HADS, confirmed in the study, justify the use of these scales to assess the emotional state and detect depressive disorders in pregnant and postpartum women. Systematic screening can identify risk factors for prenatal and postpartum depression tendencies. Mental health care for women during pregnancy and the postpartum period requires management procedures.

### Clinical Implications

1. The high prevalence of pregnancy and postpartum depression in the population of Polish women indicates the need for regular screening during antenatal and postnatal care by midwives to identify groups at risk of developing depression in pregnancy and the puerperium and to include this group of women in psychoeducation.
2. Develop a chart of risk factors for the development of pregnancy and postpartum depression based on empirical studies, which would be useful to midwives for early identification of patients with mental disorders.
3. There is a need for training (education) of medical personnel, with a particular focus on midwives caring for pregnant women with, among other things, the recognition of symptoms of depression and the use of screening tools.

## Figures and Tables

**Table 1:** The α-Cronbach coefficient of the EPDS, BDI-II, HADS-A, and HADS-D scales in the study groups.

Psychometric Scale	Pregnancy (*n* = 154)	Postartum (*n* = 163)
EPDS	0.922	0.877
BDI-II	0.907	0.926
HADS-A	0.801	0.806
HADS-D	0.798	0.839

EPDS, Edinburgh Postnatal Depression Scale; BDI-II, Beck Depression Inventory—Second Edition; HADS-A, Hospital Anxiety and Depression Scale assessing anxiety; HADS-D, Hospital Anxiety and Depression Scale assessing depression.

**Table 2:** The risk of depressive disorders depending on the psychometric (self-assessment) scale used and the adopted threshold values of indicators (scores).

Scale	Score Range	Pregnancy (*n* = 154)	Postartum (*n* = 163)	*p* *
EPDS	0–9	80 (51.95%)	108 (66.26%)	*p* = 0.03
	10–13	34 (22.08%)	28 (17.18%)	
	≥14	40 (25.97%)	27 (16.56%)	
BDI-II	0–11	78 (50.65%)	116 (71.17%)	*p* = 0.001
	12–19	38 (24.68%)	23 (14.11%)	
	20–25/@	18 (11.69%)	4 (2.45%)	
	≥26/@	20 (12.99%)	20 (12.27%)	
HADS-A	0–7	80 (51.95%)	100 (61.35%)	*p* = 0.22
	8–10	38 (24.68%)	35 (21.47%)	
	11–14/@	28 (18.18%)	21 (12.88%)	
	≥15/@	8 (5.19%)	7 (4.29%)	
HADS-D	0–7	90 (58.44%)	127 (77.91%)	*p* = 0.0001
	8–10	34 (22.07%)	27 (16.56%)	
	11–14/@	22 (14.29%)	3 (1.84%)	
	≥15/@	8 (5.19%)	6 (3.68%)	

* *p* for X^2^ test with the Yates correction; /@—categories for statistical analysis combined into one.

**Table 3:** Relative risk (RR) values for the causal category of those factors for which a statistically significant relationship was obtained in univariate analyses in the “Pregnancy” group (the RR estimated value, 95% confidence interval (95% CI), and the result of the significance test are given).

Category	EPDS	BDI-II	HADS-A	HADS-D
	Disorders Index ≥10 Points	Disorders Index ≥12 Points	Disorders Index ≥8 Points	Disorders Index ≥8 Points
Secondary (lower) education	RR = 1.95	RR = 1.84	RR = 1.41	RR = 1.88
	(1.45; 2.68)	(1.35; 2.51)	(1.02; 1.94)	(1.30; 2.71)
	*p* = 0.00005	*p* = 0.0001	*p* = 0.04	*p* = 0.0008
Type of professional work not working	RR = 1.36	---	RR = 1.58	RR = 1.93
	(0.95; 1.94)		(1.15; 2.19)	(1.36; 2.72)
	*p* = 0.09		*p* = 0.005	*p* = 0.0002
Material situation bad or average	RR = 1.62	RR = 1.56	RR = 1.44	RR = 1.82
	(1.19; 2.21)	(1.15; 2.10)	(1.05; 1.99)	(1.28; 2.59)
	*p* = 0.002	*p* = 0.004	*p* = 0.02	*p* = 0.0008
Number of pregnancies multiple births	---	RR = 1.44	---	---
		(1.06; 1.60)		
		*p* = 0.02		
Course of pregnancy complicated	---	---	RR = 1.27	---
			(0.84; 1.94)	
			*p* = 0.26	
Occurrence of prolonged periods of anxiety and sadness about the current pregnancy	RR = 1.49	RR = 1.68	---	---
	(1.06; 2.10)	(1.25; 2.27)		
	*p* = 0.02	*p* = 0.0007		
Diagnosed with depressive disorder before current pregnancy	---	RR = 1.79	---	RR = 1.22
		(1.32; 2.44)		(0.67; 2.23)
		*p* = 0.0002		*p* = 0.50
Lack of support from husband/partner/family during pregnancy	RR = 2.01	RR = 1.95	RR = 2.01	RR = 2.42
	(1.54; 2.62)	(1.50; 2.53)	(1.54; 2.62)	(1.81; 3.22)
	*p* < 0.00001	*p* < 0.00001	*p* < 0.00001	*p* < 0.00001

--- for *p* > 0.05.

**Table 4:** Relative risk (RR) values for the causal category of those factors for which a statistically significant relationship was obtained in univariate analyses in the “Postpartum” group (the RR estimated value, 95% confidence interval (95% CI), and the result of the significance test are given).

Category	EPDS	BDI-II	HADS-A	HADS-D
	Disorders Index ≥10 Points	Disorders Index ≥12 Points	Disorders Index ≥8 Points	Disorders Index ≥8 Points
Secondary (lower) education	RR = 1.16	RR = 1.18	RR = 0.99	---
	(0.75; 1.78)	(0.73; 1.91)	(0.68; 1.46)	
	*p* = 0.51	*p* = 0.50	*p* = 0.98	
Type of professional work not working	RR = 1.84	---	RR = 1.71	RR = 1.31
	(1.22; 2.78)		(1.18; 2.48)	(0.71; 2.42)
	*p* = 0.004		*p* = 0.005	*p* = 0.38
Marital status Maiden	---	RR = 1.76	RR = 1.43	RR = 1.07
		(0.99; 3.12)	(0.87; 2.35)	(0.43; 2.67)
		*p* = 0.05	NS (*p* = 0.15)	*p* = 0.88
Material situation bad or average	RR = 1.74	RR = 2.44	RR = 1.42	RR = 1.45
	(1.14; 2.64)	(1.55; 3.82)	(0.95; 2.11)	(0.79; 2.66)
	*p* = 0.01	*p* = 0.0001	*p* = 0.08	*p* = 0.23
Number of pregnancies multiple births	---	---	RR = 1.52	---
			(1.01; 2.29)	
			*p* = 0.05	
Course of pregnancy complicated	RR = 1.76	RR = 2.65	---	---
	(1.16; 2.67)	(1.64; 4.28)		
	*p* = 0.008	*p* = 0.00007		
Psychiatric treatment before current pregnancy	RR = 1.86	---	RR = 2.14	---
	(0.88; 3.94)		(1.32; 3.47)	
	*p* = 0.11		*p* = 0.002	
Occurrence of prolonged periods of anxiety and sadness about the current pregnancy	RR = 2.60	RR = 3.14	RR = 2.23	RR = 3.69
	(1.76; 3.85)	(2.08; 4.74)	(1.53; 3.24)	(2.19; 6.22)
	*p* < 0.00001	*p* < 0.00001	*p* = 0.00003	*p* < 0.00001
Diagnosed with depressive disorder before current pregnancy	RR = 3.35	RR = 3.51	RR = 2.49	RR = 4.89
	(2.63; 4.26)	(2.46; 5.01)	(1.82; 3.41)	(3.25; 7.34)
	*p* < 0.00001	*p* < 0.00001	*p* < 0.00001	*p* < 0.00001
Lack of support from husband/partner/family during pregnancy	RR = 2.83	RR = 2.84	RR = 2.04	RR = 1.14
	(1.99; 4.02)	(1.76; 4.58)	(1.30; 3.20)	(0.33; 3.92)
	*p* < 0.00001	*p* = 0.00002	*p* = 0.002	*p* = 0.85

--- for *p* > 0.05.

## Data Availability

Data are archived at the Faculty of Health Sciences in Katowice, Medical University of Silesia in Katowice. If necessary, contact the first author by e-mail umarkowska@sum.edu.pl.
